# Supplementary material for: magpie: A power evaluation method for differential RNA methylation analysis in N6-methyladenosine sequencing
Source: PLoS Comput Biol. 2024 Feb 12;20(2):e1011875. doi: 10.1371/journal.pcbi.1011875 (PMC10890765; doi:10.1371/journal.pcbi.1011875)
Supplement: S1 Appendix — (PDF) [file pcbi.1011875.s001.pdf]

# *magpie*: a power evaluation method for differential RNA methylation analysis in N6-methyladenosine sequencing

# S1 Appendix

Zhenxing Guo[&], Daoyu Duan[&], Wen Tang, Julia Zhu, William S. Bush, Liangliang Zhang, Xiaofeng Zhu, Fulai Jin and Hao Feng[*]

& These authors contributed equally to this work.

## 1 Simulation Settings

To demonstrate the power evaluation with our proposed framework, we based our simulations on samples from a GEO dataset (GSE114150) [1]. The data is obtained using the MeRIP-seq technique from eight major fetal tissues, revealing m6As related to tissue-specific activities. For DMR analysis, replicates from two experimental conditions are expected, therefore we incorporate liver and kidney samples to construct a 3 versus 3 design. Estimating parameters from this real data, we conduct 100 simulations on 10,000 candidate regions under different settings: replicates per group (2, 3, 5, 7, 10), FDR thresholds (0.05, 0.1, 0.15, 0.2), and factors of (0.3, 0.5, 0.7, 1, 3, 5, 7) proportional to the initial sequencing depth. Evaluation metrics are subsequently averaged for each scenario. We also employ four other pilot datasets (GSE120024, GSE46705, GSE47217, GSE48037) [2–5]for additional examinations.

# 2 Additional Results

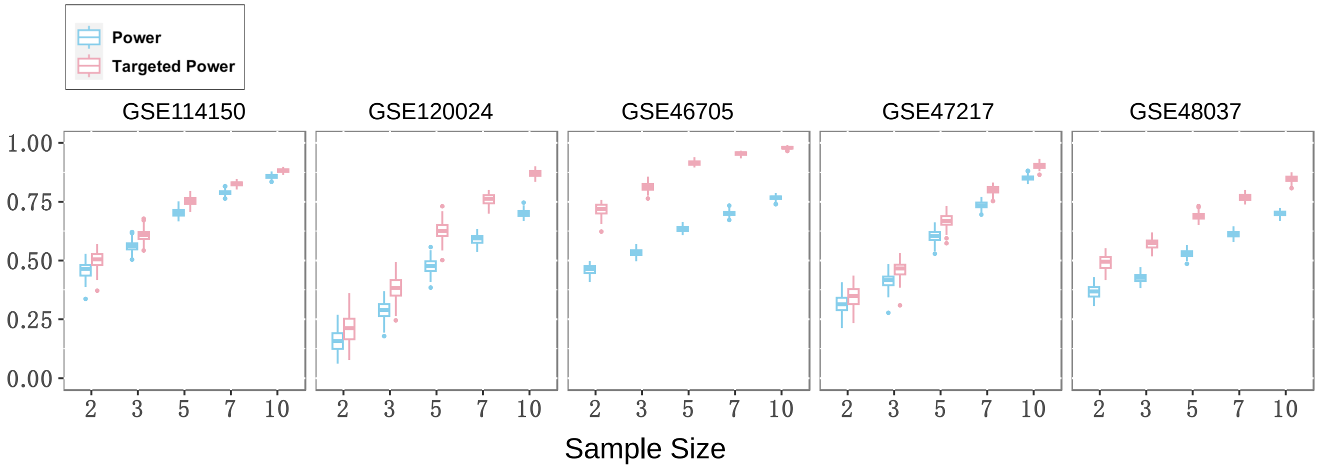


Fig A: Comparison between power and targeted power. Boxplots comparing power and targeted power ($\Delta = 2$) across different sample sizes and pilot datasets. A nominal FDR value of 0.05 is used to define significance. N=100 simulations are conducted.

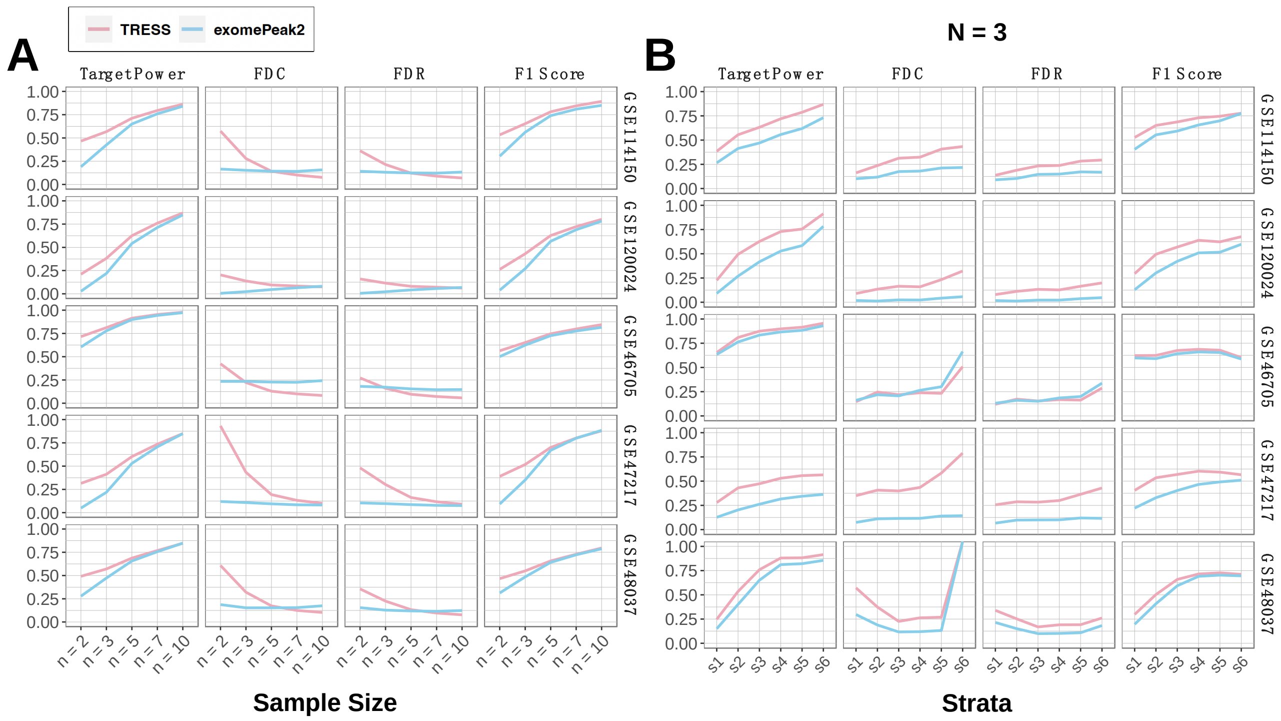


Fig B: Comparing evaluation metrics across different pilot datasets. **A** Targeted power, FDC, FDR, and F score under sample size of 2, 3, 5, 7, 10 per group, across five pilot datasets. **B** The same metrics as **A**, but only for N = 3 and stratified by mean input counts. A nominal FDR value of 0.05 is used to define significance. N=100 simulations are conducted.

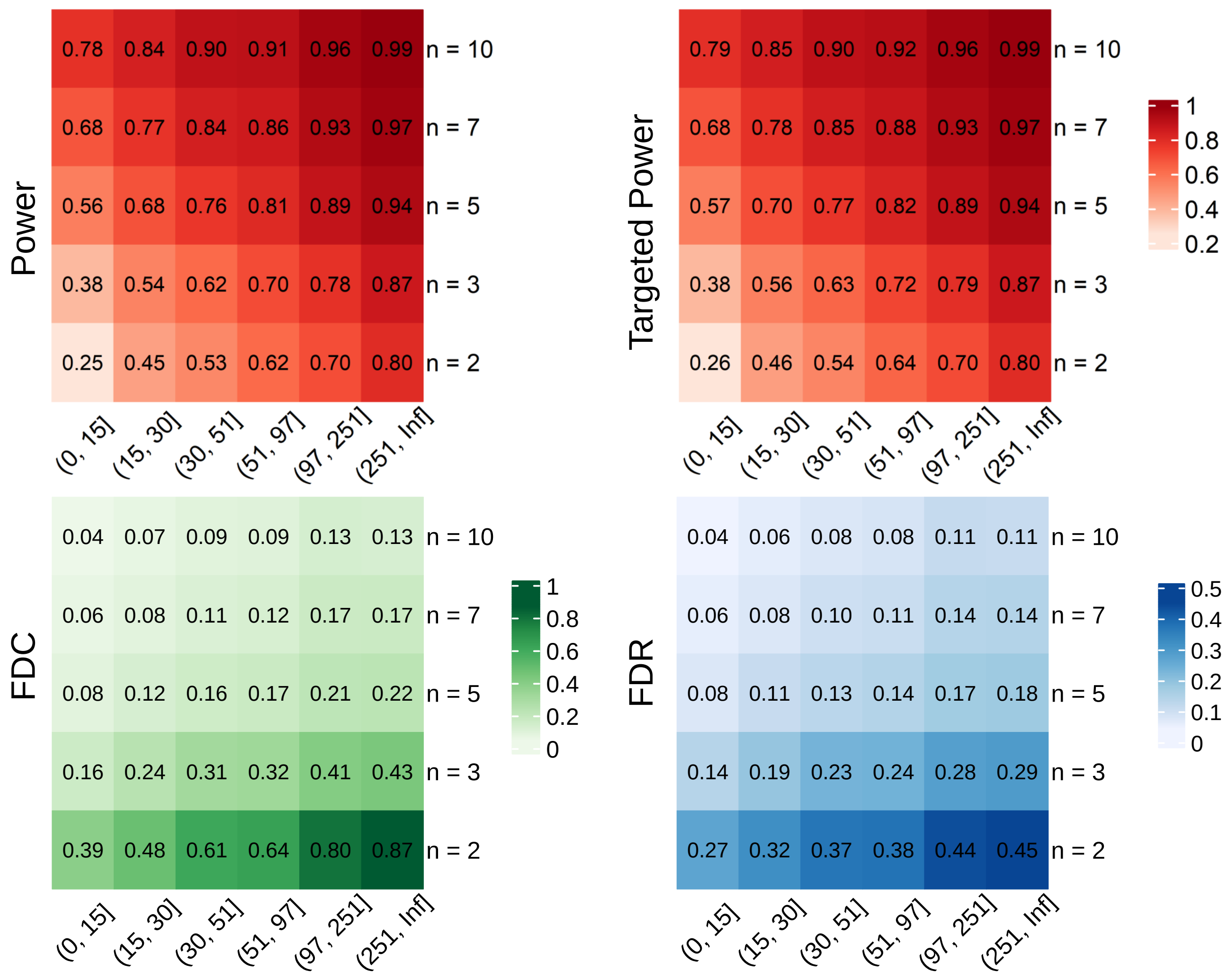


Fig C: Heatmap showing power, targeted power, FDC and FDR stratified by mean input values. Six strata are defined based on input count data quantiles: stratum 1 (0%, 10%), stratum 2 (10%, 30%), stratum 3 (30%, 50%), stratum 4 (50%, 70%), stratum 5 (70%, 90%), and stratum 6 (90%, 100%). A nominal FDR value of 0.05 is used to define significance. N=100 simulations are conducted.

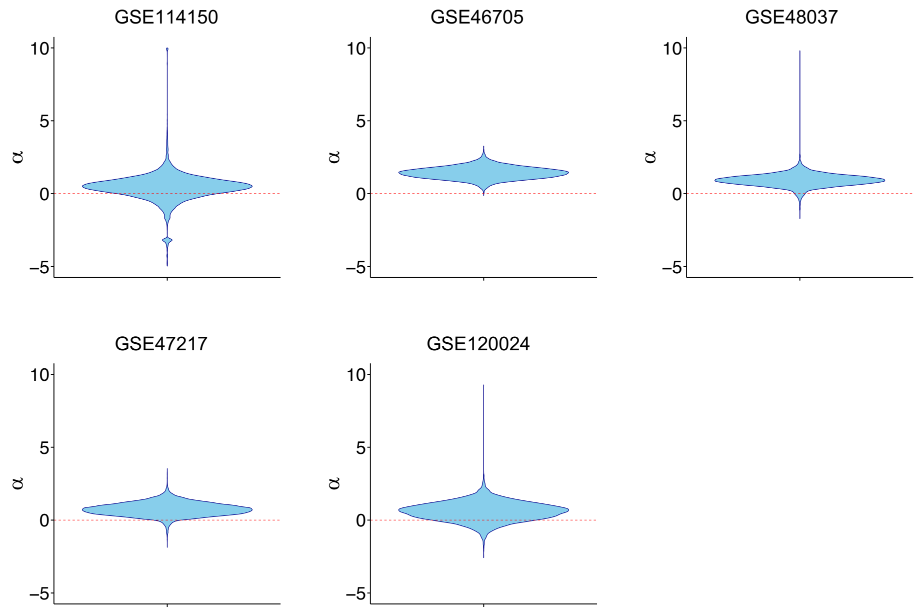


Fig D: Global distribution of the baseline expression ($\alpha_i$'s) adjusted in our model. $\alpha_i$'s are estimated from datasets GSE114150, GSE46705, GSE48037, GSE47217, GSE120024.

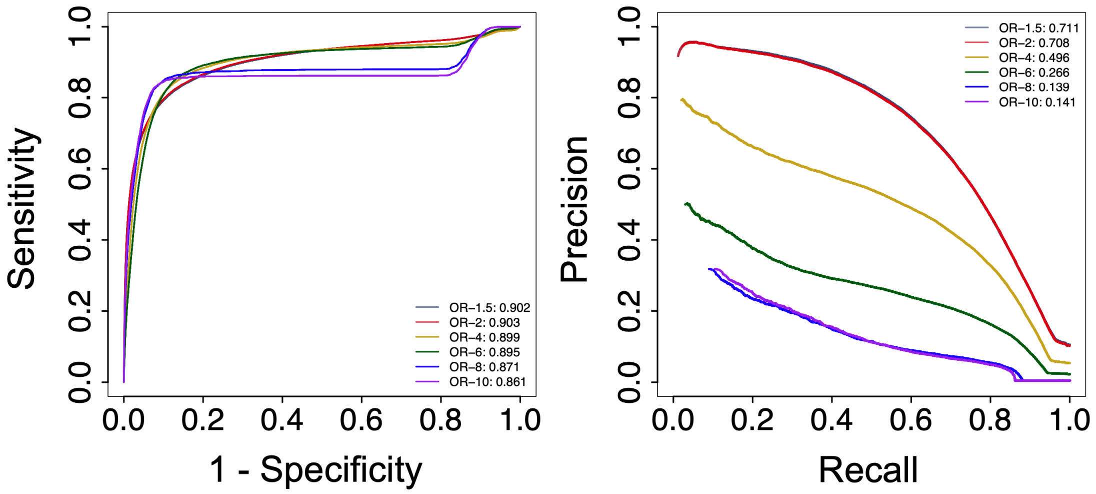


Fig E: ROC curves and Precision-recall curves under different ORs. Simulations are conducted under the scenario of 3 replicates per group, using DMR detection method TRESS. N=100 simulations are performed.

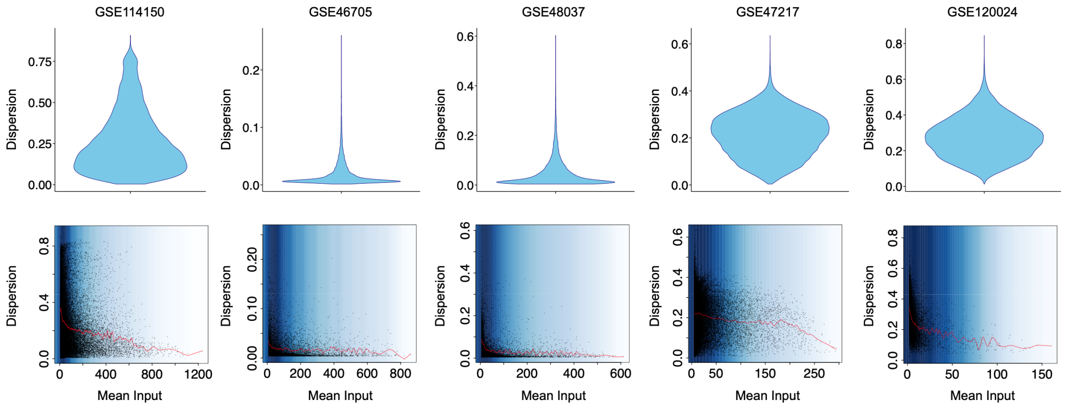


Fig F: Violin plot and Scatter plot showing the Mean-Dispersion relationship in datasets GSE114150, GSE46705, GSE48037, GSE47217, GSE120024. Mean Input values are averaged across all samples. The red line represents the fitted smooth spline.

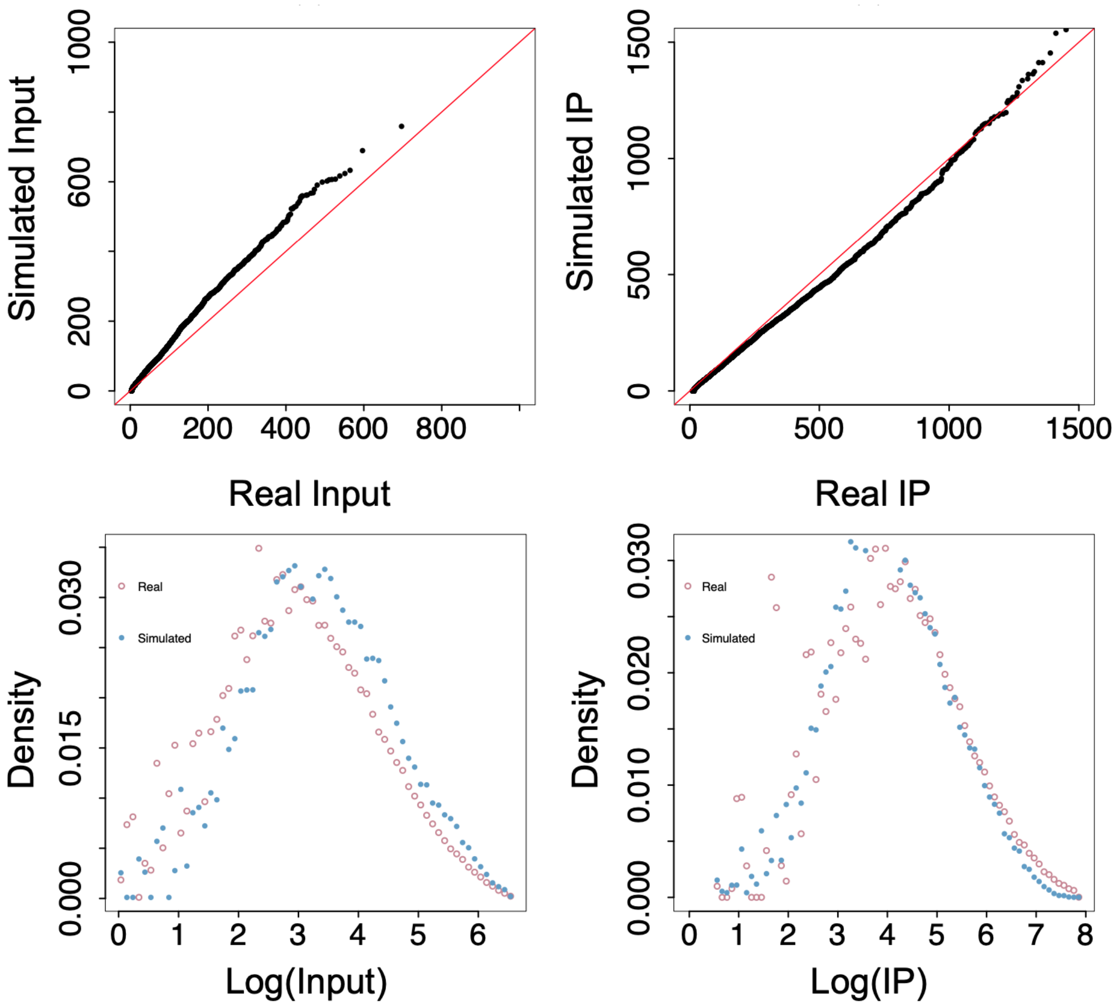


Fig G: Q-Q plot and density plot to validate simulated read counts against read counts from real data. N = 3 replicates are simulated for both groups.

# Reference

1. S. Xiao, S. Cao, Q. Huang, L. Xia, M. Deng, M. Yang, G. Jia, X. Liu, J. Shi, W. Wang, Y. Li, S. Liu, H. Zhu, K. Tan, Q. Luo, M. Zhong, C. He, L. Xia, "The RNA N6-methyladenosine modification landscape of human fetal tissues," *Nature cell biology*, vol. 21, no. 5, pp. 651–661, May 2019. https://pubmed.ncbi.nlm.nih.gov/31036937/ DOI: 10.1038/S41556-019-0315-4.

2. D. F. De Jesus, Z. Zhang, S. Kahraman, N. K. Brown, M. Chen, J. Hu, M. K. Gupta, C. He, K. Kulkarni, "m6A mRNA Methylation Regulates Human $\beta$-Cell Biology in Physiological States and in Type 2 Diabetes," *Nature metabolism*, vol. 1, no. 8, pp. 765–774, Aug. 2019. https://pubmed.ncbi.nlm.nih.gov/31867565/ DOI: 10.1038/S42255-019-0089-9.

3. Y. Niu, X. Zhao, Y. S. Wu, M. M. Li, X. J. Wang, and Y. G. Yang, "N6-methyl-adenosine (m6A) in RNA: an old modification with a novel epigenetic function," *Genomics, proteomics & bioinformatics*, vol. 11, no. 1, pp. 8–17, Feb. 2013. https://pubmed.ncbi.nlm.nih.gov/23453015/ DOI: 10.1016/J.GPB.2012.12.002.

4. M. E. Hess, S. Hess, K. D. Meyer, L. A. W. Verhagen, L. Koch, H. S. Brönneke, M. O. Dietrich, S. D. Jordan, Y. Saletore, O. Elemento, B. F. Belgardt, T. Franz, T. L. Horvath, U. Rüther, S. R. Jaffrey, P. Kloppenburg, and J. C. Brüning, "The fat mass and obesity associated gene (Fto) regulates activity of the dopaminergic midbrain circuitry," *Nature neuroscience*, vol. 16, no. 8, pp. 1042–1048, Aug. 2013. https://pubmed.ncbi.nlm.nih.gov/23817550/ DOI: 10.1038/NN.3449.

5. J. M. Fustin, M. Doi, Y. Yamaguchi, H. Hida, S. Nishimura, M. Yoshida, T. Isagawa, M. S. Morioka, H. Kakeya, I. Manabe, and H. Okamura, "RNA-methylation-dependent RNA processing controls the speed of the circadian clock," *Cell*, vol. 155, no. 4, pp. 793, Nov. 2013. https://pubmed.ncbi.nlm.nih.gov/24209618/ DOI: 10.1016/J.CELL.2013.10.026.
